# Differences in microRNA expression during tumor development in the transition and peripheral zones of the prostate

**DOI:** 10.1186/1471-2407-13-362

**Published:** 2013-07-29

**Authors:** Jessica Carlsson, Gisela Helenius, Mats G Karlsson, Ove Andrén, Karin Klinga-Levan, Björn Olsson

**Affiliations:** 1 Systems Biology Research Centre – Tumor Biology, School of Life Sciences, University of Skövde, Skövde, Sweden; 2 Systems Biology Research Centre – Bioinformatics, School of Life Sciences, University of Skövde, Skövde, Sweden; 3 Department of Laboratory Medicine, Örebro University Hospital, Örebro, Sweden; 4 Department of Urology, Örebro University Hospital, Örebro, Sweden; 5 School of Health and Medical Sciences, Örebro University, Örebro, Sweden; 6 Transdisciplinary Prostate Cancer Partnership (ToPCaP), Örebro University hospital, Clinical research centre (KFC) M-building 1st floor, Örebro 701 85, Sweden

**Keywords:** Prostate zones, Prostate cancer, MiRNA expression

## Abstract

**Background:**

The prostate is divided into three glandular zones, the peripheral zone (PZ), the transition zone (TZ), and the central zone. Most prostate tumors arise in the peripheral zone (70-75%) and in the transition zone (20-25%) while only 10% arise in the central zone. The aim of this study was to investigate if differences in miRNA expression could be a possible explanation for the difference in propensity of tumors in the zones of the prostate.

**Methods:**

Patients with prostate cancer were included in the study if they had a tumor with Gleason grade 3 in the PZ, the TZ, or both (n=16). Normal prostate tissue was collected from men undergoing cystoprostatectomy (n=20). The expression of 667 unique miRNAs was investigated using TaqMan low density arrays for miRNAs. Student’s t-test was used in order to identify differentially expressed miRNAs, followed by hierarchical clustering and principal component analysis (PCA) to study the separation of the tissues. The ADtree algorithm was used to identify markers for classification of tissues and a cross-validation procedure was used to test the generality of the identified miRNA-based classifiers.

**Results:**

The t-tests revealed that the major differences in miRNA expression are found between normal and malignant tissues. Hierarchical clustering and PCA based on differentially expressed miRNAs between normal and malignant tissues showed perfect separation between samples, while the corresponding analyses based on differentially expressed miRNAs between the two zones showed several misplaced samples. A classification and cross-validation procedure confirmed these results and several potential miRNA markers were identified.

**Conclusions:**

The results of this study indicate that the major differences in the transcription program are those arising during tumor development, rather than during normal tissue development. In addition, tumors arising in the TZ have more unique differentially expressed miRNAs compared to the PZ. The results also indicate that separate miRNA expression signatures for diagnosis might be needed for tumors arising in the different zones. MicroRNA signatures that are specific for PZ and TZ tumors could also lead to more accurate prognoses, since tumors arising in the PZ tend to be more aggressive than tumors arising in the TZ.

## Background

Prostate cancer is the most common cancer in men in Western countries and is the second leading cause of cancer death in this part of the world [1]. The prostate is divided into three glandular zones, the peripheral zone (PZ), the transition zone (TZ), and the central zone. It also has a non-glandular zone called the anterior fibromuscular stroma. The rates of cancer occurrence differ markedly between the zones, with most cancers arising in the PZ (70-75%) and in the TZ (20-25%), while only about 10% arise in the central zone. It has also been suggested that cancers in the TZ are less aggressive and have a lower biochemical recurrence rate than cancers that develop in the PZ [2,3]. Finding specific molecular signatures for tumors arising in the PZ or the TZ could potentially lead to more accurate prognoses for patients with prostate cancer.

During the last decade microRNAs (miRNAs) have been shown to be involved in cancer development, with differential miRNA expression between normal and malignant samples observed in all human cancers investigated to date [4].The diagnostic possibilities with miRNAs have increased since the discovery that miRNA expression can be measured not only in tissues but also in serum, plasma and urine [5-9]. The possibility to measure the expression of miRNAs in body fluids makes them ideal candidates for diagnostic tests and also for monitoring disease progression, such as in active surveillance. Several attempts to find miRNA expression profiles for diagnosis and prognosis of prostate cancer have been made during the last years, but the results have been inconclusive since different miRNAs have been implicated in each profile suggested to date. The results nevertheless indicate that it is possible to find a set of miRNA markers for diagnosis and prognosis of prostate cancer, since all studies resulted in sets of miRNAs which could separate between normal and malignant prostate tissues [10-17]. However, a caveat is that none of these studies reported from which prostatic zone the samples were taken. Therefore, one limiting factor for the diagnostic/prognostic value of the candidate miRNA biomarkers may be the differences in miRNA expression patterns between the zones, both in normal and malignant prostate tissues. This could further partly explain the lack of agreement between the miRNA sets identified in the different studies.

Currently, little is known about the differences in gene and protein expression between the prostate zones, but it seems reasonable to assume that the preference for cancer development in a specific zone is caused by pre-existing transcriptome differences between the three zones in normal tissue. These assumed pre-existing differences could in part be due to developmental differences of the zones, since the peripheral and transition zones develop from the endoderm of urogenital sinus while the central zone develops from the wolffian duct [18,19]. Two large-scale studies have elucidated the differences in mRNA expression between the zones in normal prostate tissue. Noel *et al*. analysed 24,325 genes and reported that 43 of these were differentially expressed between PZ and TZ in normal tissues [20]. Heul-Nieuwenhuijsen *et al*. investigated 15,000 genes and found 346 of these to be differentially expressed between PZ and TZ [21], with only five genes overlapping with the results of the study by Noel *et al*. This large difference in the number of differentially expressed genes, as well as the small overlap, could be due to differences between the materials used in the two studies as well as between the analysis methods.

The results of the two above mentioned studies [20,21] indicate that there are differences in gene expression between the two zones, and the precise nature of these differences needs to be investigated further. It is also noteworthy that no studies have been performed regarding miRNA expression in normal prostate tissue. Furthermore, neither mRNA nor miRNA expression has been compared between malignant tissues from the different zones. The aim of the present study was to explore the miRNA expression patterns in different zones of the prostate, both in normal and malignant tissue, and to investigate the relationship between miRNA expression and incidence of cancer in the PZ and TZ.

## Methods

### Patient material

The COSM cohort (Cohort of Swedish Men) was established in the Västmanland and Örebro counties of Sweden in 1997. It includes 48,850 men born between 1918 and 1952. Until December 2009, 3232 men in the cohort had been diagnosed with prostate cancer, 300 of which had subsequently been subjected to radical prostatectomy. Complete follow up is available for all men with prostate cancer until January 2011. In order to get a homogenous study material where potential differentially expressed miRNAs reflect differences in zone expression, rather than differences in tumor aggressiveness, patients were only included in the study if they had a Gleason grade 3 tumor in the PZ, the TZ, or both. From the 300 men subjected to radical prostatectomy, 13 patients having a tumor with Gleason grade 3 in the TZ (n=5), in the PZ (n=5) or in both (n=3) were included in the study. From the latter three patients, one sample of malignant tissue was taken from each zone. We also included normal prostate tissue from 10 patients diagnosed with bladder cancer, who had been subjected to radical cystoprostatectomy (sample 1N-10N in Table 1). The included normal prostate tissue was examined by a pathologist after radical cystoprostatectomy with the same procedure as after a radical prostatectomy and assessed for prostate cancer without any findings. From each bladder cancer patient, two samples of normal prostate tissue were collected, one from the TZ and one from the PZ (Table 1). The study was approved by The regional ethical review board in Uppsala, Sweden (2009/016, Written informed consent for participation in the study was obtained from the participants as well as consent to publish the data in Table 1).

**Table 1:** Description of patient material included in the study

**Patient**	**Age**	**PZ (GS)**	**TZ (GS)**	Death*	**PSA (ng/mL)**
1N	80	-	-	0	-
2N	79	-	-	0	-
3N	76	-	-	0	-
4N	66	-	-	0	-
5N	66	-	-	0	-
6N	53	-	-	0	-
7N	52	-	-	0	-
8N	45	-	-	0	-
9N	70	-	-	0	-
10N	71	-	-	0	-
11M	67	3	NT	0	-
12M	71	3	NT	-	8
13M	79	3	NT	0	26
14M	73	3	3	0	-
15M	57	3	NT	0	-
16M	77	3	3	0	-
17M	76	3	NT	0	-
18M	63	NT	3	0	8
19M	65	3	3	0	-
20M	74	NT	3	1	5
21M	91	NT	3	1	8
22M	78	NT	3	1	32
23M	79	NT	3	1	7

N = Normal prostate sample from cystoprostatectomy.

M = Malignant prostate sample from radical prostatectomy.

PZ (GS) = Gleason score in peripheral zone.

TZ (GS) = Gleason score in transition zone.

NT = No tumor in this zone.

* 1= Dead, 0 = Alive.

- Data not available.

### miRNA profiling

A pathologist marked the PZ and TZ in both normal and tumor areas on formalin fixed paraffin embedded (FFPE) prostate tissues, and three cores (Ø0.6 mm) were collected from each tissue for usage in subsequent total RNA extraction. The expression profiling was performed as previously described [10]. In short, total RNA was extracted using the RecoverAll total nucleic acid isolation kit optimized for FFPE tissues (Ambion) before reverse transcription using the TaqMan® MicroRNA reverse transcription kit and Megaplex™ RT primers, human pool v2.0 (Applied Biosystems). The cDNA samples were pre-amplified using Megaplex™ PreAmp primers and TaqMan® Preamp master mix (Applied Biosystems) and then diluted in a 0.1X TE Buffer (pH 8.0) before use in the qPCR reaction. The diluted pre-amplified cDNA was mixed with TaqMan® PCR master mix II (No AmpErase UNG, Applied Biosystems) and run in a 40 cycle qPCR reaction on the TaqMan® MicroRNA A and B Cards version 2.0, thus measuring the expression of 667 unique miRNAs (Applied Biosystems). All reactions were performed on the Applied Biosystems 7900 HT system.

### Data analysis

Raw C_T_-values were calculated using the SDS software (Applied Biosystems), applying manually selected thresholds for each miRNA. Normalization and computation of statistical tests was performed in the programming software R [22]. The data were normalized using qPCRNorm quantile normalization [23]. A paired Student’s t-test (*p<*0.05) was used to identify miRNAs that were differentially expressed between the TZ and PZ in normal tissues, whereas the corresponding unpaired t-test was used for identifying miRNAs that were differentially expressed between normal and malignant tissues in each zone, as well as for the comparison between malignant tissues from the different zones (Additional file 1). Results are reported both with and without correction of the *p*-values for multiple testing, using the Benjamini-Hochberg method.

Hierarchical clustering was performed on all samples and miRNAs investigated using the PermutMatrix clustering tool [24], using Euclidean distance when comparing expression profiles and the average linkage rule when comparing clusters. Expression values were normalized using the mean center columns method in the clustering software. Differentially expressed miRNAs were also clustered using the same method as well as used in a principal component analysis using Omics Explorer, version 2.3 (Qlucore AB, Lund, Sweden).

For the 15 miRNAs with lowest *p*-values for differential expression between normal PZ and TZ, experimentally validated target genes were extracted from TarBase [25] and miRecords [26] while predicted target genes for the same miRNAs were extracted from MicroCosm targets [27]. These target genes were then compared to genes previously identified as differentially expressed between normal TZ and PZ in the prostate, to investigate if there was an overlap [20,21]. Experimentally validated target genes were also extracted for miRNAs identified as differentially expressed between normal and malignant TZ and PZ tissues using the same databases [25,26] and pathway analysis was performed on the validated target genes using the DAVID functional annotation tool [28].

The ADTree algorithm in the WEKA data mining tool was used to identify zone-specific signatures, in the form of alternating decision trees [29,30], for classification of tissues. The generality of the identified signatures for classification of unseen tissues was estimated using the leave-one-out cross-validation procedure [31].

## Results

In this study we included 13 patients from the Cohort of Swedish men (COSM), which had been diagnosed with prostate cancer and subjected to a radical prostatectomy. The patients had tumors with Gleason grade 3 in the TZ (n=5), in the PZ (n=5) or in both (n=3), from the latter three patients, one sample of malignant tissue was taken from each zone. Normal prostate tissue from ten patients diagnosed with bladder cancer and subjected to a radical cystoprostatectomy was also included in the study (Table 1). The expression of 667 unique miRNAs was analyzed using the TaqMan**® **MicroRNA array set v2.0 from Applied Biosystems.

**Figure 1:**
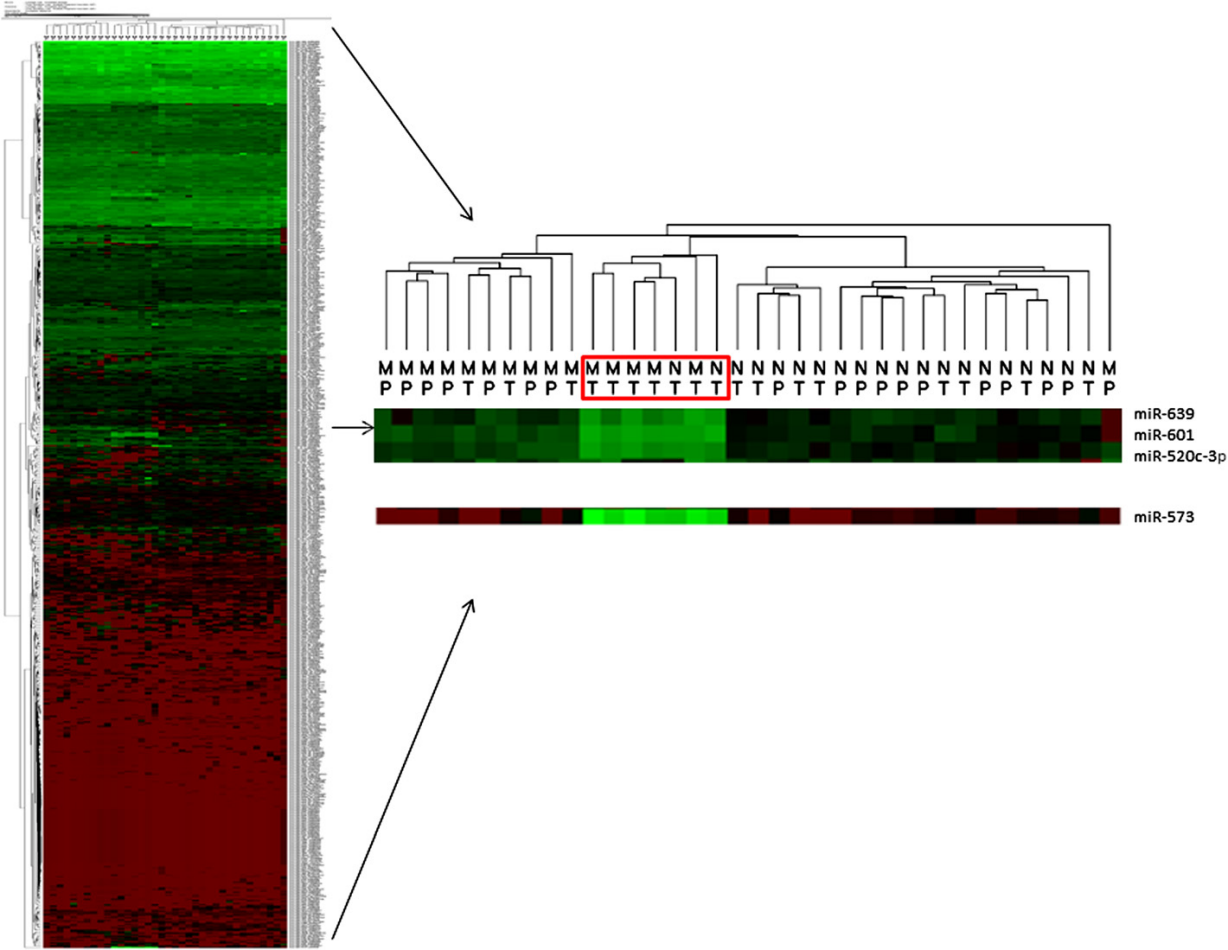
**Clustering of all miRNAs and all samples investigated.** Clustering of all 667 miRNAs and all 36 samples investigated and the specific expression of five miRNAs in seven of the samples from the TZ. Normal samples are labeled N and malignant samples are labeled M. Samples from the TZ are labeled T and samples from the PZ are labeled P. Green colors are high expression values while red colors are low expression values.

Hierarchical clustering was performed on all samples and all miRNAs investigated in the study (Figure 1). All samples except for two normal samples could be separated between normal and malignant tissues indicating that the expression profiles of all 667 miRNAs investigated can be used to separate between these two types of tissues. There is also a tendency for the tissues of the peripheral zone to cluster together and the tissues from the transition zone to cluster together, regardless of malignancy state. One of the clusters, which include five malignant and two normal samples from the transition zone, had very specific expression profiles of four miRNAs (hsa-miR-639, hsa-miR-601, hsa-miR-520c-3p and hsa-miR-573), separating them from the rest of the samples (Figure 1).

**Figure 2:**
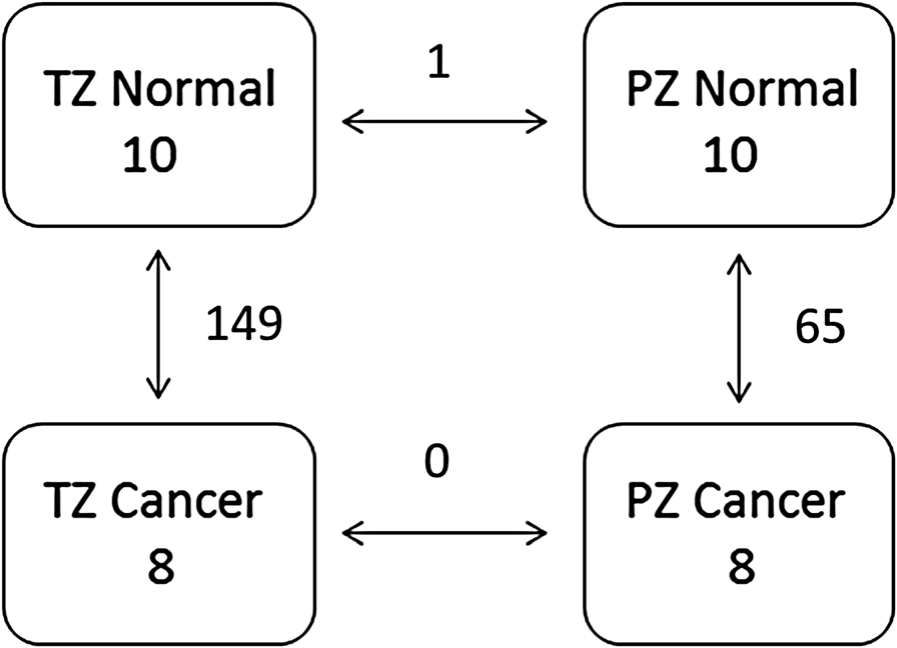
The number of differentially expressed miRNAs found between all combinations of sample groupings.

Student’s t-tests were performed, with and without correction for multiple testing, on all combinations of sample groupings, i.e. normal TZ tissue vs. normal PZ tissue, malignant TZ tissue vs. malignant PZ tissue, normal TZ tissue vs. malignant TZ tissue, and normal PZ tissue vs. malignant PZ tissue (for complete results see Additional files 2 and 3). The largest sets of differentially expressed genes were found in the comparisons between normal and malignant tissues (Figure 2). Between normal and malignant tissues from the TZ 149 miRNAs were found to be significantly differentially expressed (231 before applying correction for multiple testing). The same comparison in the PZ identified 65 significantly differentially expressed miRNAs (150 before correction). In contrast, only a single miRNA was significantly differentially expressed between the TZ and PZ in normal tissue (51 before correction) and none between the TZ and PZ in malignant tissue (50 before correction). Overall, these numbers clearly indicate that the main differences in miRNA expression occur between normal and malignant tissues, rather than between the prostate zones, and that these differences arise during tumor development. However, the particular miRNAs that are differentially expressed in tumor tissues vs. normal tissues may very well be different for the different zones.

**Figure 3:**
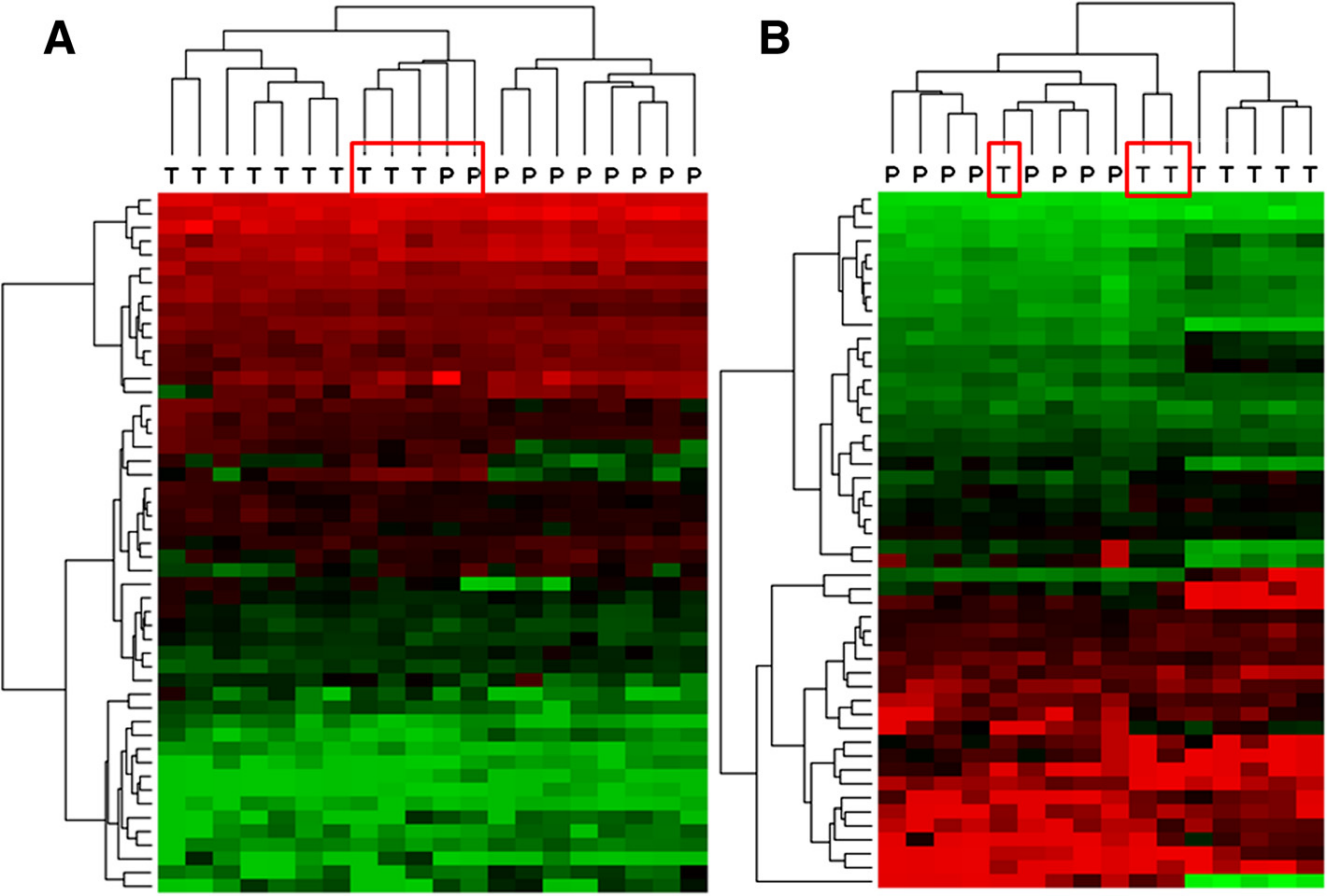
**Clustering’s on differentially expressed miRNAs between TZ and PZ tissues.** Clustering’s are based on miRNAs found to be differentially expressed (before multiple testing adjustment) between TZ and PZ samples from normal tissue **(A)** and malignant tissue **(B)**. The clustering of normal samples resulted in three major clusters, one with seven TZ samples, one with eight PZ samples, and one mixed cluster containing three TZ and two PZ samples (marked with red box). The clustering of malignant samples resulted in two major clusters, of which one was mixed (i.e. contained three misplaced TZ samples, red boxes) and one was a small homogeneous TZ cluster. Green colors are high expression values while red colors are low expression values.

**Figure 4:**
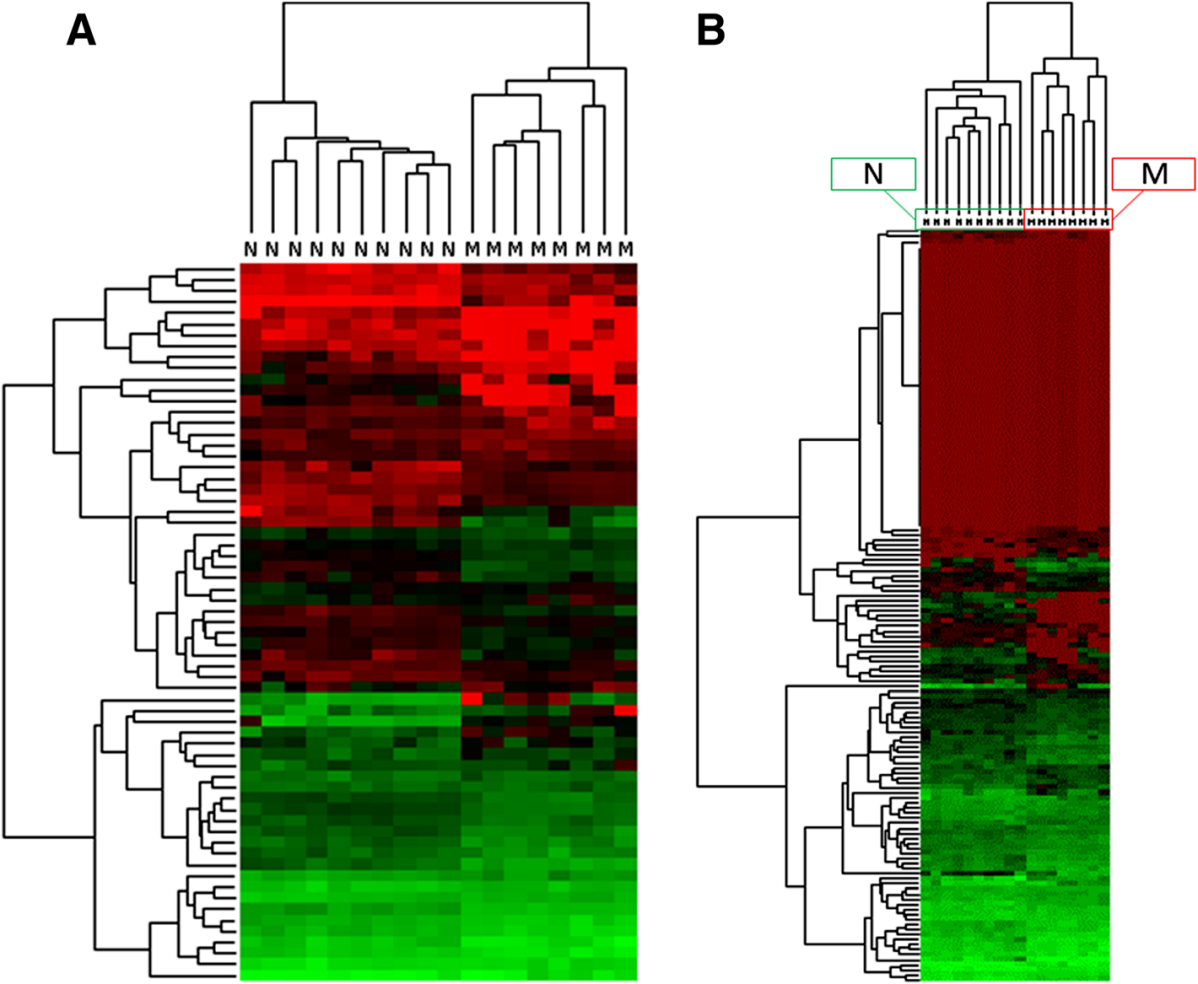
**Clustering’s on differentially expressed miRNAs between normal and malignant tissues.** Clustering’s are based on miRNAs found to be differentially expressed (after multiple testing adjustment) between normal and malignant tissue in the PZ **(A)** and in the TZ **(B)**. Each clustering resulted in two major clusters, which were both homogeneous with respect to normal and malignant tissues. Green colors are high expression values while red colors are low expression values.

The miRNAs identified as differentially expressed before multiple testing adjustments in the comparison between normal TZ vs. normal PZ and malignant TZ vs. malignant PZ were used in the subsequent analyses. For the comparison between normal vs. malignant TZ and normal vs. malignant PZ, only miRNAs identified as differentially expressed after adjustment were used. The differentially expressed miRNAs were used in hierarchical clustering and principal component analyses (PCA). Overall, the clusterings based on miRNAs differentially expressed between TZ and PZ showed several misplaced samples, whereas the clusterings based on miRNAs differentially expressed between normal and malignant samples showed perfect separations of the sample groups into two major clusters (Figures 3 and 4). Similarly, the PCA results showed unclear separation between TZ and PZ tissues (Additional file 4) and a much clearer separation between normal and malignant tissues (Additional file 5).

**Figure 5:**
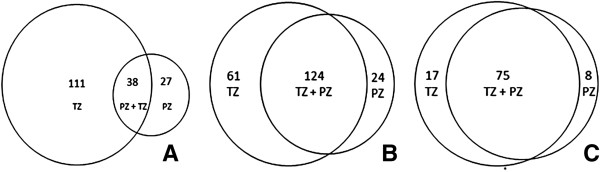
**Venn diagram showing the results of target gene and pathway analyses.** Venn diagram showing the overlap between **A)** Differentially expressed miRNAs in normal and malignant tissues in TZ and PZ, **B)** Overlap of validated target genes for miRNAs found to be differentially expressed between normal and malignant TZ vs. PZ and **C)** Overlap of pathways for the validated target genes. The overlaps were 22%, 59% and 75%, respectively.

The 65 miRNAs that were differentially expressed between normal and malignant PZ tissues were subsequently compared to the 149 miRNAs that were differentially expressed between normal and malignant TZ tissues. The comparison revealed that 111 (75%) of the miRNAs differentially expressed in the TZ were unique for TZ but only 27 (42%) of the miRNAs differentially expressed in the PZ were unique for the PZ (Figure 5A). To further investigate the similarities between these miRNA sets, validated target genes for the miRNAs were extracted from miRecords and TarBase [25,26]. A comparison of the target genes for miRNAs differentially expressed in PZ and TZ showed that TZ and PZ tumors had 124 target genes in common (59%), while only 61 (29%) and 24 (12%) target genes where specific for the TZ and PZ tumors, respectively (Figure 5B).Additionally, a pathway analysis was performed on the validated target genes, using the DAVID functional annotation tool. This resulted in 100 different pathways of which 75 (75%) were common for the TZ and PZ, 17 (17%) were specific for the TZ target genes and 8 (8%) were specific for the PZ target genes (Figure 5C and Additional files 6 and 7). Specific pathways for the TZ included pathways for infection and inflammation responses and PTEN-dependent cell cycle arrest, while specific pathways for the PZ included cell cycle control, Dicer pathway, TGF-beta signaling pathway and Wnt signaling pathway.

The 15 miRNAs with lowest *p*-values for differential expression between TZ and PZ in normal tissue were chosen for a more detailed target gene analysis. Validated and predicted target genes for these 15 miRNAs were extracted from TarBase, miRecords and MicroCosm and subsequently compared to mRNA genes previously identified as differentially expressed between PZ and TZ in normal prostate tissues [20,21]. The results show that all these miRNAs have predicted target genes which have previously been identified as differentially expressed between the two zones, while only two of the miRNAs (miR-181c and miR-127-3p) have validated target genes that have been described as differentially expressed in previous studies (Table 2). Of the differentially expressed genes between normal TZ and PZ found by Noel *et al.* and Van der Heul-Nieuwnhuijsen *et al*, 21% and 29%, respectively, were in this study found to be target genes for the 15 miRNAs with lowest *p*-values for differential expression between TZ and PZ in normal prostate tissue.

**Table 2:** **The 15 miRNAs with lowest *p*-values for differential expression between TZ and PZ in normal prostate tissue and their previously identified differentially expressed target genes**

**miRNA**	**+/−**	**Predicted (validated) target**	**Noel *et al*. **[20]	**Van der Heul-Nieuwenhuijsen *et al*. **[21]
		**genes**		
miR-433	+	880 **(2)**	*	NAT1, SRPX, KLK3, EDN3, KIAA1324
miR-494	+	707 **(1)**	DHX9, SYTL2	PCOLCE, LUM, SRPX, RAB27A, MED28, PCP4, CCL2
miR-22*	+	696 **(0)**	*	BAMBI, PFKFB3, RAB10, NEXN, ATF3, FOLH1, CEBPD, SNX25, MED28, LRRC28
miR-15a*	-	664 **(0)**	*	PFKFB3, EGR2, EGR1, BIK, KLK3,
				PBEF1, TRPM4, DDX5
miR-15b*	-	669 **(0)**	*	NEXN, SRPX, EGR1, HAT1, PBEF1,
				UAP1
miR-379	+	799 **(1)**	S100A4	C6orf115, GMNN, RBP1, CALD1, ASPA, DBI, ATP2C1, SERPINI1, SRPX, DUSP1, ECM1, RABGGTB, GRP58, TM4SF1, CCL2
miR-216b	-	862 **(0)**	HSD11B1	EAF2, C6orf115, IFNGR1, TFPI2,
				HAT1, PENK, PBEF1, LPHN2, SFRS9
miR-181c	-	990 **(5)**	HSD11B1	**GATA6**, SFRS5, KCNMA1, FKBP1A, ZFYVE26, KLF6, PRKAG2, THBS4, LPHN2, SPOCK3, EIF4A2, TACSTD2, MYBPC1, TGBR3, RAB3IP, TUBB
miR-543	+	875 **(0)**	*	KCNMA1, C6orf115, DACH1, FKBP1A. PRKAG2, SERPINI1, RABGGTB, THBS4, PTGS2, TM4SF1, EIF4A2,SFRS9, KIAA1324, RAB3IP, CCL2
miR-27b*	+	722 **(0)**	HSD11B1	ASPA, MLPH, FBXO2, BRI3, PRRX2, PENK, UAP1, SGCE, TRIM36, EIF4A2, TBCA
miR-154	+	738 **(0)**	EFEMP1	EAF2, TNFSF10, GMNN, CSRP2,
				GCAT, SRPX, FEZ1, SGCE, DDX5
miR-424*	+	535 **(1)**	DHX9, HOXD13	HEPH, C6orf115, FKBP1A, PRKAG2, HPN, ECM1, THBS4, COL16A1, CCL2
miR-495	+	896 **(0)**	*	SFRS5, TACSTD1, C6orf115, JUNB, SERPINI1, MCM2, NANS, DUSP1, CITED2, LIMS2, NIPA2, NDN, PBEF1, TFF1, UAP1, RPRM, CYP1B1, RAB3IP, CCL2
miR-337-3p	+	888 **(0)**	*SPON1*	NRG2, TRIT1, ATF3, *SPON1*, CSRP2,
				C15orf5, COL16A1, NDN, LHFP
miR-127-3p	+	741 **(0)**	C6orf32, NELL2	**XBP1**, KCNMA1, FKBP1A, GCAT, GPR30, KCNJ8, TUBGCP2, TFF1, PTGDS, GADD45G

+/− Up/down-regulated in the transition zone compared to the peripheral zone.

* No target gene (validated or predicted) overlap.

Genes marked in bold are validated target genes.

Italicized genes have been found to be differentially expressed between PZ and TZ in both reference papers [20,21].

To evaluate potential markers for PZ and TZ tissues a classification procedure was performed using the ADTree algorithm, which generates trees where each decision node specifies a miRNA and a threshold expression value, while the prediction nodes contain numbers, which are summed up when the classification is done. AD trees were repeatedly generated and tested using the leave-one-out cross validation procedure and the classification accuracy was defined as the percentage of correctly classified test samples. The results from the cross-validation correspond with the results from the clustering, PCA and Student’s t-test, showing that the major differences lie between normal and malignant tissues rather than between the two zones. For classification of normal and malignant tissues, an accuracy of 100% (PZ) and 94% (TZ) was reached and the AD trees contained only two miRNAs (Table 3). For classification of normal TZ and PZ tissues, an accuracy of 70% was reached and the AD tree contained six miRNAs, while for malignant TZ and PZ tissues, only 56% accuracy was reached and the AD tree contained eight miRNAs (Table 3).

**Table 3:** Results from the cross-validation procedure for evaluation of potential markers

**Comparison**	**Accuracy**	**No. of miRNAs**	**miRNA names**
Normal PZ vs. Malignant PZ	100%	2	miR-187 and miR-19a
Normal TZ vs. Malignant TZ	94%	2	miR-143 and miR-25
Normal TZ vs. Normal PZ	70%	6	miR-93, miR-95, miR-154, miR
			541, miR-539, miR-28-3p
Malignant TZ vs. Malignant PZ	56%	8	miR-145-3p, miR-19b-1-5p, miR-493-5p, miR-195, miR-548b-5p, miR-182-3p, miR-95, miR-187

## Discussion

To our knowledge, this is the first time that miRNA expression patterns have been analysed and compared between the PZ and the TZ of both normal and malignant prostate tissues. Unique miRNA signatures for tumors arising in the PZ and TZ could be beneficial in the diagnosis of prostate cancer if they reflect significant differences between tumors of different origin. Signatures for tumors of different origin could also help in making more accurate prognoses, since tumors arising in the PZ are suggested to be more aggressive and associated with worse outcome. Today, there are no specific expression signatures (neither mRNA- or miRNA-based) for the different prostatic zones. Prostate tumors often consist of several independent foci and it is difficult to identify the original focus and where it arose, since a tumor can arise in one zone and grow into the adjacent zone.

When performing a hierarchical clustering of all samples and miRNAs investigated in this study, we found a cluster of seven TZ samples, with an expression profile specific for four miRNAs. There is no obvious reason for this phenomenon regarding clinical data since the cluster includes both normal and malignant tissues (two normal and five malignant). To our knowledge, only one of these five miRNAs (miR-520c) has been implicated in prostate cancer before [32,33]. In these studies, miR-520c was down regulated in prostate cancer tissues and it was suggested that it is involved in tumor migration and invasion, thus constituting a metastasis-promoting miRNA [33]. This does not agree with our results since miR-520c is upregulated in malignant tissues compared to normal tissues, regardless of zonal origin. Included in this study are four patients who died from their prostate cancer and two of the samples from these patients are found within this specific cluster. Since miR-520c is considered to be a metastasis-promoting miRNA this leads to the hypothesis that the set of four miRNAs somehow could be related to a more aggressive disease. However, this does not explain why two normal samples were included in the cluster or why the other two samples with a bad outcome of their prostate cancer were not included. Further studies need to be performed to investigate the expression of these four miRNAs in a larger cohort to be able to explain the reason for this differential expression between TZ tissues.

One miRNA, miR-433, was significantly differentially expressed between normal PZ and TZ tissues in this study. This miRNA has two validated target genes, *HDAC6* and *FGF20*, which have both been implicated in tumor development [34-36]. High levels of HDACs results in increased proliferation, decreased apoptosis, increased angiogenesis and induction of different oncogenes [37]. *FGF20 *is normally only expressed in the adult central nervous system but is expressed in malignant tissues [38], and therefore it seems reasonable to think that *FGF20* is under strong control of miR-433 in normal prostate tissues and that this control is lost during tumor progression. Since miR-433 is over-expressed in normal TZ tissue compared to normal PZ tissue, it could be hypothesized that the up-regulated miR-433 suppresses its target genes, *HDAC6* and *FGF20*, and results in extra protection against tumor development in the TZ, and that this function is not found in the normal PZ. This hypothesis could be a possible explanation for the difference in tumor occurrence between the zones. Van der Heul-Nieuwenhuijsen *et al*., has a similar hypothesis for the PZ. They found that genes that are over-expressed in normal PZ tissue also tend to be over-expressed in PZ tumors. They suggested that this high expression of genes in normal PZ could support malignant growth, thus making the PZ more prone to tumor development [21].

Since only one miRNA was found to be significantly differentially expressed between normal PZ and TZ tissues, the 15 miRNAs that were closest to a statistically significant differential expression were chosen for target gene analysis. This analysis showed that two of the validated target genes (XBP1 and GATA6) and 107 predicted target genes (see Table 2) have been found to be differentially expressed in previous studies [20,21]. This result indicates that a substantial proportion of the deregulated mRNA expression is due to deregulated miRNA expression, since 21% of the mRNA genes identified in (15) and 29% of the mRNA genes identified in (16) are target genes of the 15 miRNAs included in this analysis.

A central issue in this work is to discern where the major differences in miRNA expression occur, between the zones of the prostate, or between normal and malignant tissues. Many more differentially expressed miRNAs were found when comparing normal with malignant tissue (149 for TZ tissues, and 65 for PZ tissues) than when comparing tissues from the different zones (only one miRNA for normal TZ vs. normal PZ, and none for malignant TZ vs. malignant PZ). This strongly indicates that the major differences in the transcription program are those arising during tumor development, rather than during normal tissue development. At the same time, the clustering and principal component analysis indicate that also the non-significant changes in miRNA expression between tissues from the two zones are large enough for detection of zonal origin (TZ or PZ). It is important to keep in mind that many small, but coordinated, changes in expression can be significant when considered in combination, even if the changes in expression of the individual miRNAs are statistically non-significant.

The results from the AD tree classification procedure showed that normal and malignant tissues could be classified with an accuracy of 100% (PZ) and 94% (TZ) with only two miRNAs used in the tree. One miRNA, miR-187, appears in the ADtree for classification of normal vs. malignant PZ tissues as well as for malignant TZ vs. malignant PZ tissues. This indicates that miR-187 can generally be used to classify tumors arising in the PZ. The same scenario is seen for miR-95, which appears in the ADtree for normal TZ vs. normal PZ and malignant TZ vs. malignant PZ, indicating that miR-95 can be used to classify TZ vs. PZ tissues in different scenarios. None of these miRNAs have validated target genes although they have been found to be deregulated in cancer in previous studies. MiR-187 has been found to be upregulated in ovarian cancers and was also associated with recurrence-free survival and could be used as an independent prognostic factor for ovarian cancer [39]. MiR-95 has been shown to promote cell growth in colorectal cancer cells [40]. The hypothesis that these two miRNAs can be used to classify between normal and malignant PZ tissues (miR-187) and between TZ and PZ tissues (miR-95) needs to be validated in a new, larger material.

When comparing the lists of differentially expressed miRNAs between normal and malignant TZ and PZ it was found that the TZ had more unique differentially expressed miRNAs (111) compared to the PZ (27) (Figure 5). This indicates that the changes during tumor development are more extensive in the TZ compared to the PZ since the changes in the TZ involve more miRNAs of which many are unique for the TZ. These results show that there may be a need for zone-specific marker sets for diagnosis and prognosis. In the target gene and pathway analysis we could see that even though there is a large overlap between target genes and pathways in TZ and PZ, there are still unique genes and pathways for each zone. This further strengthens the indication that there are differences in how tumor development occurs in the different zones. It should be noted that the target gene and pathway analysis was only performed on validated target genes. Different results could be found if predicted target genes were also included in the analysis.

One limitation of this study is its size, since only 10 normal samples from each zone and eight malignant samples from each zone were included. This could in part explain the lack of statistically significant differentially expressed miRNAs between normal TZ and PZ samples and malignant TZ and PZ samples. It is also possible that there is no difference between normal TZ and PZ and that the difference is found in how the tumor develops, although one would expect to find a difference between malignant TZ and PZ samples since we have shown that different miRNAs are differentially expressed between normal and malignant tissues in TZ and PZ. A second limitation of this study is the limited histo-pathological data. This study could be seen as an initial attempt, indicating on which miRNAs the focus should lie in future studies to further elucidate the differences in miRNA and/or mRNA expression between TZ and PZ zones, both in normal and malignant tissues.

## Conclusions

The results of this study indicate that the major differences in the transcription program are those arising during tumor development, rather than during normal tissue development. In addition, tumors arising in the TZ have more unique differentially expressed miRNAs compared to the PZ. The results also indicate that separate miRNA expression signatures for diagnosis might be needed for tumors arising in the different zones.

## Abbreviations

CT: Cycle threshold; CZ: Central zone; FFPE: Formalin fixed paraffin embedded; miRNA: MicroRNA; nt: nucleotide; PZ: Peripheral zone; qPCR: Quantitative polymerase chain reaction; TE: Tris EDTA; TZ: Transition zone.

## Competing interests

The authors declare that they have no competing interests.

## Authors’ contributions

All authors participated in the design of the study. JC carried out all laboratory work, performed all data analyses and wrote the initial draft of the manuscript. GH, OA, KKL and BO supervised the project. MK carried out the pathological marking of the tissues. JC, BO and KKL jointly improved the manuscript from the initial draft. JC and BO analysed the clusterings, PCA and AD tree results. All authors read and approved the final manuscript.

## Pre-publication history

The pre-publication history for this paper can be accessed here:

http://www.biomedcentral.com/1471-2407/13/362/prepub

## Supplementary Material

Additional file 1 **Overview of the sample sets and comparisons of expression levels.** PZ normal and TZ normal samples are paired (two samples from the same patient), whereas normal and malignant samples from each zone are unpaired, as well as the malignant samples from different zones (which were taken from different prostate cancer patients). Click here for file

Additional file 2 **Differentially expressed miRNAs (*p*<0.05) between normal and malignant TZ tissues before multiple testing adjustments.** Click here for file

Additional file 3 Differentially expressed miRNAs (p<0.05) between normal and malignant PZ tissues before multiple testing adjustments. Click here for file

Additional file 4 **Principal component analysis on differentially expressed miRNAs between PZ and TZ tissues.** The principal component analysis is based on the miRNAs found to be differentially expressed (before multiple testing adjustment) between PZ and TZ in normal prostate samples (**A**) and malignant tissue samples (**B**). Green = PZ, Red = TZ. Click here for file

Additional file 5 **Principal component analysis on differentially expressed miRNAs between normal and malignant tissues.** The principal component analysis is based on the miRNAs found to be differentially expressed (after multiple testing) between normal and malignant PZ tissues (**A**) and normal and malignant TZ tissues (**B**). Green = Malignant, Red = Normal. Click here for file

Additional file 6 Results from pathway analysis of target genes for the differentially expressed miRNAs between normal and malignant TZ tissues. Click here for file

Additional file 7 Results from pathway analysis of target genes for the differentially expressed miRNAs between normal and malignant PZ tissues. Click here for file
