# Supplementary material for: Differences in microRNA expression during tumor development in the transition and peripheral zones of the prostate
Source: BMC Cancer. 2013 Jul 29;13:362. doi: 10.1186/1471-2407-13-362 (PMC3733730; doi:10.1186/1471-2407-13-362)
Supplement: Additional file 1 — Overview of the sample sets and comparisons of expression levels. PZ normal and TZ normal samples are paired (two samples from the same patient), whereas normal and malignant samples from each zone are unpaired, as well as the malignant samples from different zones (which were taken from different prostate cancer patients). [file 1471-2407-13-362-S1.pdf]

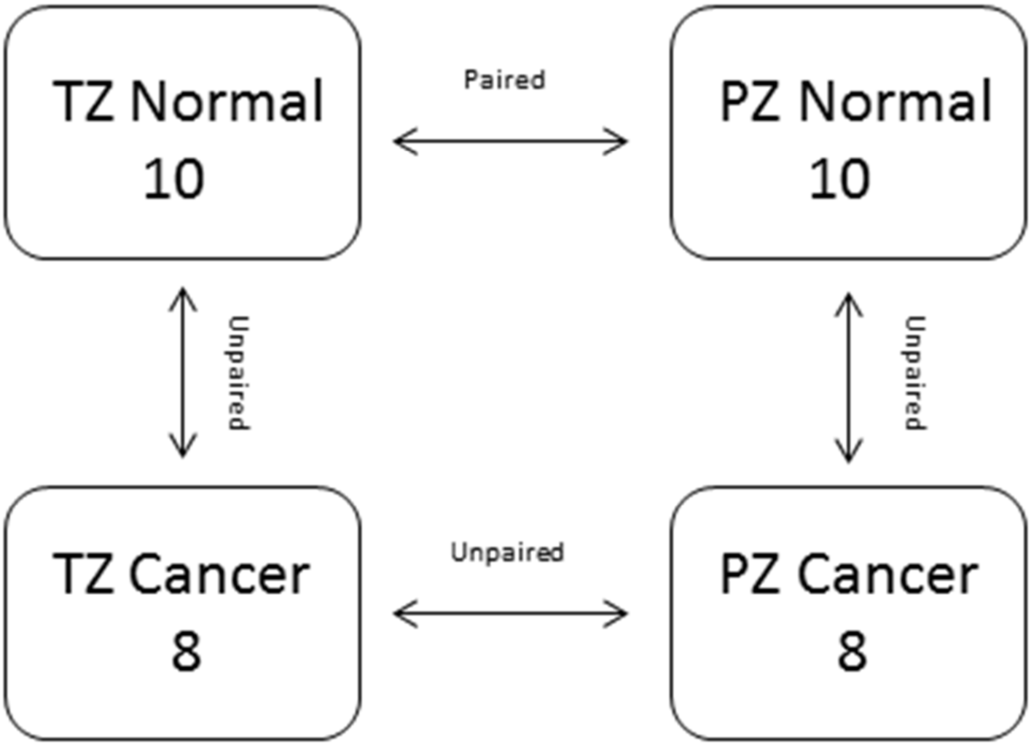

Additional file 1. Overview of the sample sets and comparisons of expression levels.

PZ normal and TZ normal samples are paired (two samples from the same patient), whereas normal and malignant samples from each zone are unpaired, as well as the malignant samples from different zones (which were taken from different prostate cancer patients).
