# Supplementary material for: Differences in microRNA expression during tumor development in the transition and peripheral zones of the prostate
Source: BMC Cancer. 2013 Jul 29;13:362. doi: 10.1186/1471-2407-13-362 (PMC3733730; doi:10.1186/1471-2407-13-362)
Supplement: Additional file 4 — Principal component analysis on differentially expressed miRNAs between PZ and TZ tissues. The principal component analysis is based on the miRNAs found to be differentially expressed (before multiple testing adjustment) between PZ and TZ in normal prostate samples (A) and malignant tissue samples (B). Green = PZ, Red = TZ. [file 1471-2407-13-362-S4.pdf]

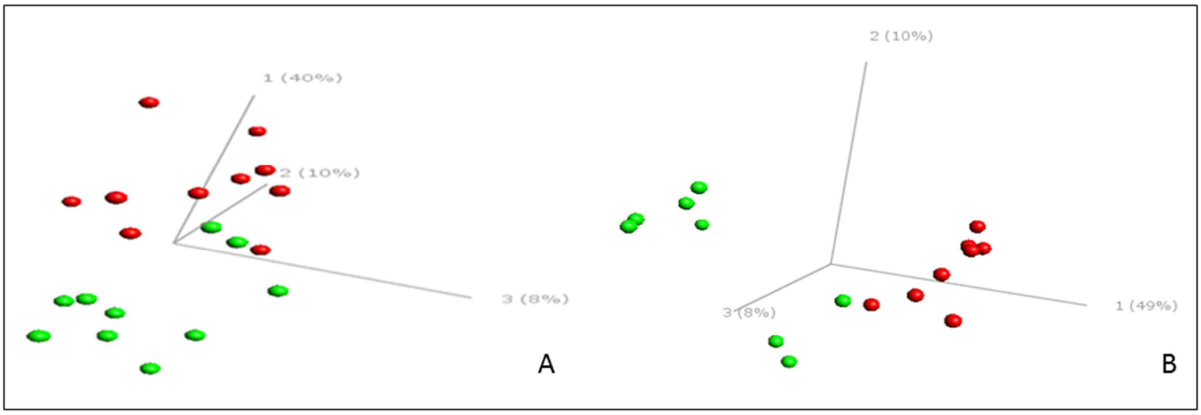

Additional file 4. Principal component analysis on differentially expressed miRNAs between PZ and TZ tissues.

The principal component analysis is based on the miRNAs found to be differentially expressed (before multiple testing adjustment) between PZ and TZ in normal prostate samples (A) and malignant tissue samples (B). Green = PZ, Red = TZ
