# Supplementary material for: Differences in microRNA expression during tumor development in the transition and peripheral zones of the prostate
Source: BMC Cancer. 2013 Jul 29;13:362. doi: 10.1186/1471-2407-13-362 (PMC3733730; doi:10.1186/1471-2407-13-362)
Supplement: Additional file 5 — Principal component analysis on differentially expressed miRNAs between normal and malignant tissues. The principal component analysis is based on the miRNAs found to be differentially expressed (after multiple testing) between normal and malignant PZ tissues (A) and normal and malignant TZ tissues (B). Green = Malignant, Red = Normal. [file 1471-2407-13-362-S5.pdf]

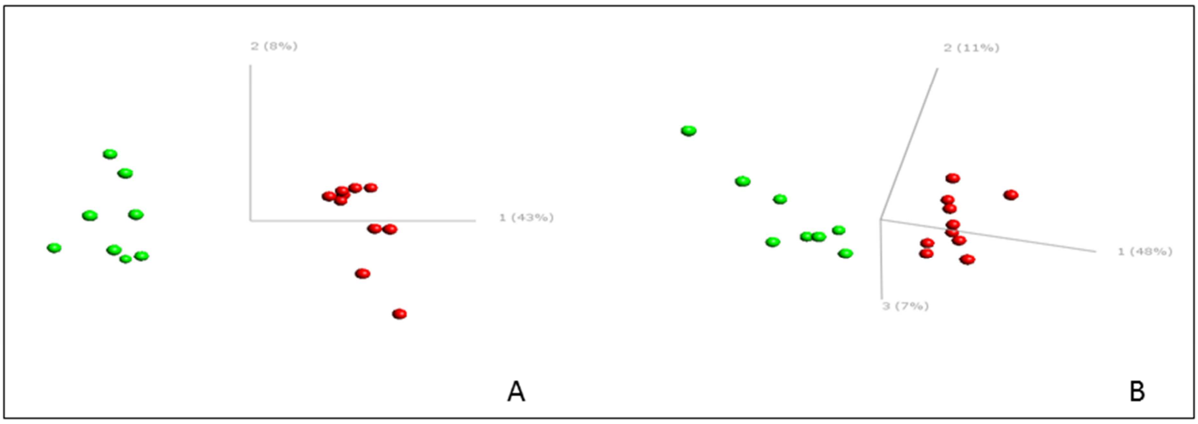

Additional file 5. Principal component analysis on differentially expressed miRNAs between normal and malignant tissues.

The principal component analysis is based on the miRNAs found to be differentially expressed (after multiple testing) between normal and malignant PZ tissues (A) and normal and malignant TZ tissues (B). Green = Malignant, Red = Normal.
